# Chiroptical activity of gas-phase propylene oxide predicting the handedness of interstellar circular polarization in the presolar nebula

**DOI:** 10.1126/sciadv.add4614

**Published:** 2022-11-18

**Authors:** Adrien D. Garcia, Jérémie Topin, Jana Bocková, Nykola C. Jones, Søren V. Hoffmann, Cornelia Meinert

**Affiliations:** ^1^ Institut de Chimie de Nice, Université Côte d’Azur, UMR 7272 CNRS, Nice 06108, France.; ^2^ ISA, Department of Physics and Astronomy, Aarhus University, 8000 Aarhus, Denmark.

## Abstract

Propylene oxide, the first chiral molecule recently detected in the interstellar medium, has once again raised the question whether biomolecular chirality might have cosmic origins. However, accurate chiroptical properties of propylene oxide in the ultraviolet spectral range necessary to suggest possible asymmetric synthetic routes in the gas phase are scarce. Here, we report on the first experimental measurements of the anisotropy spectra of gas-phase propylene oxide in the vacuum ultraviolet spectral range. Our experimental results provide novel insights into the handedness of interstellar circular polarization at the dawn of molecular evolution of our star- and planet-forming region. Besides the fundamental importance of this new investigation for understanding the origin and evolution of homochirality on Earth, our high-resolution experimental electronic circular dichroism data will inspire new efforts in quantum computational spectroscopy.

## Teaser

Left-handed stellar circular polarization may have enriched the *R*-enantiomer of propylene oxide in meteorites.

## INTRODUCTION

Propylene oxide, also known as 2-methyl oxirane, has recently regained considerable attention being the first chiral molecule detected outside of our Solar System (*1*). Chiral molecules are extremely important in chemistry, physics, biology, and drug discovery (*2*–*8*). Despite their variable stereoselectivity in various chemical reactions, the homochiral nature of all chiral biomolecules is a decisive prerequisite for an enormous range of cellular functions (*9*). While homochirality is evolutionarily advantageous, it is still unknown how the molecular single-handedness of the building blocks of the complex trinity— DNA, RNA, and proteins—came about (*10*). Several asymmetric processes have been experimentally tested to induce chirality in molecular systems (*11*–*20*), but those focusing on circularly polarized light (CPL) appear to us to be the most encouraging, especially given the results reported on CPL-induced molecular chirality in amino acids (*21*–*27*). Chirality transfer from photons to matter and vice versa (*28*–*30*) is not only of fundamental importance to understanding one of the most puzzling questions of the early origins of life (*31*, *32*) but also of practical importance as a preparative method for synthesis of chiral compounds (*33*–*36*) or next-generation photonic devices (*37*–*39*).

Experiments that use CPL illumination to induce a chiral bias require prior knowledge of the chiroptical response of the targeted matter to tune desired enantiomeric excesses (*ee*). Circular dichroism (CD) spectroscopy, based on the difference in absorption of left and right CPL (*l*- and *r*-CPL) of chiral molecules, remains the preferred technique to study chiral discrimination, because of its straightforward operation, high accuracy, and efficiency (*40*). The intrinsic enantioselectivity toward CPL of a given handedness and wavelength can be then quantified by the so-called anisotropy or dissymmetry factor *g* = ∆ε/ε, the ratio between the differential extinction coefficient ∆ε, and the extinction coefficient ε (*41*–*43*).

**Fig. 1.**
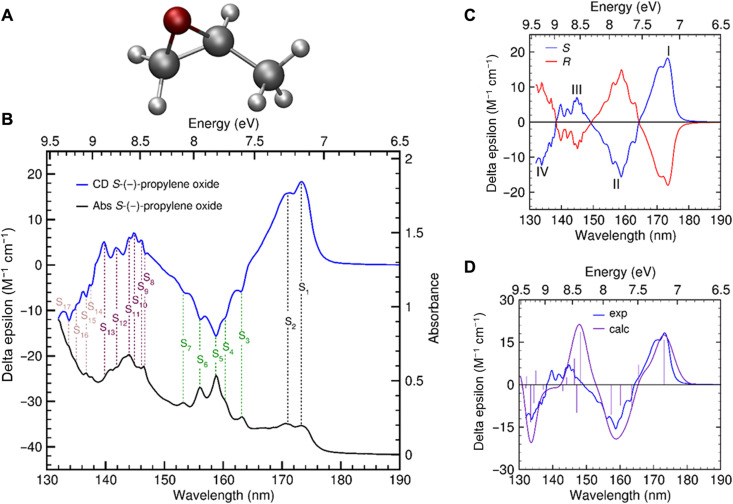
Gas-phase chiroptical properties of propylene oxide. (**A**) Optimized structure of *S*-(−)-propylene oxide (CAM-B3LYP/aug-cc-pVQZ). (**B**) Experimental CD (blue) and absorbance (black) spectra of *S*-(−)-propylene oxide. (**C**) Experimental CD spectra of *R-*(+)-enantiomer (red) and *S*-(−)-enantiomer (blue) of propylene oxide exhibiting mirror symmetry. (**D**) Experimental (blue line) and theoretical CD spectrum (purple line) of *S*-(−)-propylene oxide based on vertical excitations (TD-DFT/CAM-B3LYP and aug-cc-pVQZ); FWHM, 0.12 eV; rotatory strengths are indicated as stick bars.

Propylene oxide (Fig. 1A), a relatively simple chiral molecule, has recently been detected in the interstellar medium toward the high-mass star-forming region Sagittarius B2(N) (*1*). At the time, the observations did not allow the determination of whether there is a difference between the relative abundance of the two propylene oxide enantiomers. However, high-precision polarization measurements could provide a definite answer in the future.

The spectroscopic properties of propylene oxide and its derivatives have been extensively studied in the past (*44*–*48*). Because of the minimal conformational freedom of propylene oxide, it is, moreover, an ideal model compound for theoretical method development for chiroptical spectroscopy (*49*–*51*). However, the only low-resolution experimental CD spectra of propylene oxide previously reported in the ultraviolet (UV) (*46*) and vacuum UV (VUV) wavelength range (*44*, *45*) show only very weak and inconsistent features for the higher-lying electronic states with no mirror symmetry confirmation of relative intensities, signs, and band positions of the two enantiomers. These experimental data are thus inadequate for validation of theoretical approaches. We have recently refreshed the field of gas-phase CD and anisotropy spectroscopy with a new experimental setup that combines an entirely new gas cell with synchrotron radiation from the AU-CD beam line at ASTRID2, Aarhus (Denmark) (*52*).

Here, we report on the first experimental measurements of the anisotropy spectra of *R*-(+)- and *S*-(−)-propylene oxide along with synchrotron CD and absorption spectra in the VUV spectral region. The concerted experimental and in vacuo theoretical experiments ultimately provide structural information on the electronic and vibronic transitions as well as the oscillator and optical rotatory strengths. Quantitative models based on these newly measured anisotropy spectra revealed correlations between the *R*-(+)-enantiomer–enriched propylene oxide derivative detected in the Murchison meteorite (*53*) and the helicity of CPL in our presolar cloud with far-reaching consequences in understanding the origins of biomolecular homochirality.

## RESULTS

### Gas-phase chiroptical spectra

The anisotropy factor *g* as a function of wavelength was studied by recording simultaneously the CD ∆ε and absorption ε spectra of each enantiomer of propylene oxide using a gas of analytes in a dedicated sample cell with a length of 500 mm at the AU-CD beam line on ASTRID2 at Aarhus University (*52*). Figure 1B shows the CD and associated absorption spectra of *S*-(−)-propylene oxide along with the most dominant resolved experimental electronic and vibronic transitions highlighting the high resolution of gas-phase synchrotron CD spectroscopy. To provide accurate anisotropy data of both enantiomers, the CD (fig. S1) and absorbance spectra (fig. S2 and table S1) were measured at various gas flow rates. Individually recorded CD spectra could be scaled directly with the cell pressure during measurements to yield spectra of similar shape and magnitude. The absolute CD signal, corrected for gas density and path length, is given in units of molar CD (per molar per centimeter) often referred to as Delta epsilon units as used in Fig. 1.

CD spectroscopy of both enantiomers of propylene oxide yielded two almost perfectly mirrored spectral curves (Fig. 1C), revealing the best resolved vibrational fine structure to date (fig. S1 and table S2). *S*-(−)-propylene oxide displays the most pronounced maxima at around λ = 173 (CD band I) and 145 nm (III) and minima at around 159 (II) and 134 nm (IV), while *R*-propylene oxide shows mirror symmetry in CD signs and relative intensities.

To compute the CD spectrum of *S*-(−)-propylene oxide, the first 16 time-dependent density functional theory (TD-DFT) vertical excitations were convoluted with Gaussian broadening functions with full width at half maximum (FWHM) of 0.12 eV. Vertical excitation energies and rotatory strengths *R* computed with different functionals and basis sets are summarized in table S4 and compared with former theoretical results (table S5). Best agreement with our experimental data was found using the CAM-B3LYP functional, which was tailored explicitly to treat Rydberg-type and diffuse excitations (*54*). In combination with the aug-cc-pVQZ basis set, the convergence for the low-lying computed excitation energies and rotatory strengths is reasonably good, while larger deviation occurs for CD bands III and IV. According to previous literature (*44*, *45*, *50*, *51*, *55*), the CD transitions were assigned as the Rydberg transitions from either *n*(O), the lone pair orbital on the oxygen atom, or the σ-type orbital localized along the oxirane ring bonds (table S5, A and B). The often mixed s- and p-characters of involved Rydberg-type orbitals in combination with the fine structure of our experimental gas-phase CD spectra attributable to vibrational modes—not considered in our theoretical model—make definite electronic assignments of the experimental excitation energies rather challenging.

Apart from a spectral shift of CD band III, the sign and spectral position of the calculated CD bands match those of our measured spectrum (Fig. 1D), further supporting the consistency of our data. The discrepancies in the energy range of 8 to 9 eV obviously highlight the challenges in predicting CD spectra of oxiranes, particularly where the overall signal is a result of cancelation of vibronic transitions of opposite signs with distinct vibrational structures (*50*, *51*, *56*). Our first coherent high-resolution experimental absorbance and CD data of both propylene oxide enantiomers in the VUV spectral region, therefore, open a plethora of avenues for profound optimization of computational methods that consider vibrational modes to accurately simulate chiroptical properties.

**Fig. 2.**
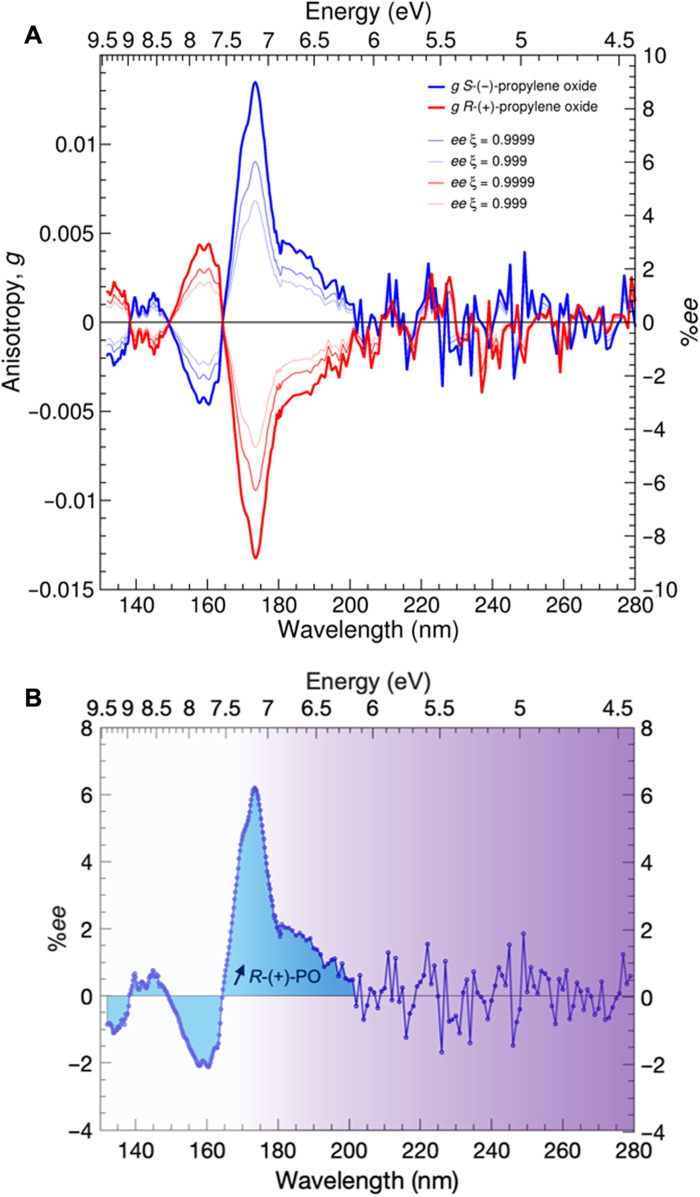
Gas-phase anisotropy spectra of propylene oxide. (**A**) Anisotropy spectra of *R*-(+)-propylene oxide (red) and *S*-(−)-propylene oxide (blue). Thin lines indicate the lower limit of the inducible enantiomeric excess (*ee*) by either *l*-CPL or *r*-CPL as a function of wavelength and extent of reaction ξ calculated on the basis of the relation reported in (*43*). (**B**) Integrated area (blue) under the %*ee* curve obtained for *S*-(−)-propylene oxide with an extent of reaction ξ = 0.9999. Purple color and brightness indicate the decline in UV photon flux of low-mass main sequence stars (*60*). Stellar broadband *l-*CPL would induce an excess in *R*-propylene oxide (PO).

The intensity of the anisotropy bands follows the intensity pattern of the CD spectra. Band I is the most intense feature in both CD and anisotropy spectra, followed by bands II, IV, and III (Fig. 2A). Consequently, the highest %*ee* can be induced in the wavelength range of 165 to 200 nm with values predicted to be about 6% for a reaction rate of 0.9999 and *g*_174nm_ = 1.3 × 10^−2^ based on the relation reported in (*43*) and recapped in the Supplementary Text. The predicted *ee* curves over the entire anisotropy wavelength range are shown as thin lines in Fig. 2A for two selected photolysis rates ξ of 0.999 (99.90%) and 0.9999 (99.99%), respectively. Further anisotropy values and their calculated minimum inducible %*ee* by asymmetric photolysis at the extent of reaction 0.9999 at energies corresponding to the extrema of anisotropy bands I to IV are given in table S3.

### Asymmetric photochemistry at the dawn of molecular cloud evolution

The enantiomeric enrichment of naturally occurring l-amino acids (*57*, *58*) and d-sugar derivatives (*59*) found in carbonaceous meteorites offers the sole example of asymmetry outside of the biosphere and has raised the hypothesis of a possible link between astrochemical evolution and the origin of homochiral biomolecules. However, both the origin of this extraterrestrial chiral bias and the emergence of the first chiral molecule itself are still unanswered. Asymmetric photochemistry by stellar CPL is arguably the most compelling explanation behind the chiral bias found in meteorites. This idea gained support when CP radiation in the near-infrared was found in the active sites of star-forming regions of the Orion Nebula (*60*–*62*) and in NGC 6334-V (*63*). Considering that a primordial chiral bias originated in the interstellar medium and was at least partially incorporated into planetesimals and planets, extraterrestrial chiral molecules could have thus contributed to the chiral choice of homochiral life on Earth. Interstellar ice simulation experiments revealed a formation mechanism of racemic propylene oxide via galactic cosmic ray–driven nonequilibrium chemistry involving suprathermal oxygen at very low temperatures and its subsequent sublimation during temperature-programmed desorption at 100 K (*64*). While the predominant photodecomposition of gas-phase propylene oxide upon UV radiation is known to proceed via a ring opening followed by a 1,2-hydrogen shift to give propanal and to a minor extent acetone (*65*), our anisotropy data predict that chiral discrimination via asymmetric photolysis of racemic propylene oxide in the gas phase could lead to an enantiomeric enrichment when the photolysis is incomplete. No chiroptical data are now available for propylene oxide in the solid state, simulating the surface of interstellar dust grains. A derivative of propylene oxide has recently been detected in the ethanol extracts of the Murchison meteorite (*53*). The enantioselective analysis revealed a large *ee_R_* of about 10%, i.e., an excess of the *R*-(+)-enantiomer.

Under realistic astrophysical conditions, natural sources of circularly polarized radiation are spectrally broadband in the pertinent UV spectral region (*60*–*62*), and photochemical activation of multiple CD/anisotropy bands of opposite signs is expected. To investigate the net effect of asymmetric photolysis of non-monochromatic CPL emission, integration of each wavelength interval corresponding to an anisotropy band and integration over the entire investigated wavelength range have been performed (Table 1 and fig. S3). The results show that broadband UV irradiation by stellar *l*-CPL with a constant flux in the measured wavelength range would induce an *ee* in favor of the *R*-enantiomer of gas-phase propylene oxide of at least 1%. The calculated net %*ee_R_* over the range of 138 to 202 nm can be thought of as a lower limit considering the reduction of UV photon flux in the far UV range (*60*), resulting in a weaker impact of the *g* bands between 132 and 164 nm compared to the most intense *g* band spreading from 164 to 202 nm. The most pronounced anisotropy band I coinciding with the dominant UV emission wavelength range of most of the stars (*60*) would thus dictate the handedness of UV CPL inducible enantiomeric excess (Fig. 2B).

**Table 1.** Inducible *ee* values for propylene oxide. Integrated anisotropy *g* bands and inducible minimum enantiomeric excess *ee_R_* through asymmetric photolysis of racemic propylene oxide (PO) at the extent of reaction ξ = 0.9999 and broadband UV *l-*CPL with uniform flux density in the UV range.

λ/nm	Average *g* (×10^−2^)*	Average %*ee_R_*^‡^	Average %*ee_R_*^§^	
164–202	0.51	0.21	2.4	1.0
149–164	−0.29		−1.4	
138–149	0.08		0.4	
132–138	−0.18		−0.8	

*Average anisotropy *g*(λ) of each active *g* band of *S*-PO.

^†^Average anisotropy *g*(λ) over the entire *g* spectra of *S*-PO.

^‡^Average inducible *ee_R_* of each active *g* band.

^§^Average inducible *ee_R_* over the entire *g* spectra.

Although we cannot exclude the impact of asymmetric solid-state photolytic reactions on the surface of icy grains, which may differ due to the influence of a surrounding polar solvent on the chiroptical activity of propylene oxide, we assume that its overall impact on the total inducible *ee* is rather limited. The lowest energy anisotropy band attributed to the *n*_O_ → *R*(3s) transition is expected to be in the far UV even in the solid state where the ice matrix dominates the stellar CPL absorption with energies above 6.5 eV. Nevertheless, future studies of the influence of a surrounding water matrix on the excitation energy and the sign of CD band I recorded in the gas phase between 7 and 7.5 eV by a surrounding water matrix are envisaged.

## DISCUSSION

**Fig. 3.**
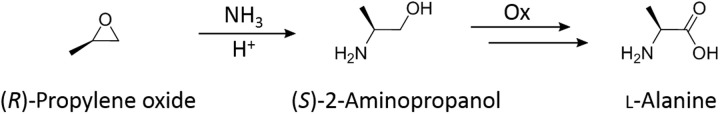
Gas synthetic pathway from *R*-(+)-propylene oxide to l-alanine. First step: Epoxide opening in acidic condition. Second step: Over-oxidation of *S*-(+)-2-aminopropanol.

If our Solar System was formed in a high-mass star-forming region, which is supported by the presence of short half-life radionuclides such as ^60^Fe and ^26^Al in primitive meteorites (*66*)—according to our anisotropy-based prediction—it may have been irradiated with left-handed CPL to result in an excess of *R*-(+)-propylene oxide and its derivatives in the Murchison meteorite. While our current prediction of the inducible %*ee_R_* in propylene oxide should be considered as the lower estimate due to the nonlinear decline of the stellar CPL in the VUV (*60*), desorption-condensation cycles connecting the solid interstellar ices with the surrounding gas may have been crucial for an *ee* amplification of interstellar propylene oxide starting from a few percent, as shown here in the gas phase, to about 10% *ee_R_* as identified in the Murchison meteorite (*53*). Besides, it is a common view that not only meteoritic amino acids but also their chiral bias may have originated from their precursor structures via Strecker-type synthesis during parent body aqueous alteration (*53*). Note that *R*-(+)-propylene oxide can, in two steps, lead to the proteinogenic amino acid l-alanine. The nucleophilic attack of ammonia (NH_3_) on the stereogenic carbon of the *R*-(+)-propylene oxide under acidic conditions leads to *S*-(+)-2-aminopropanol, the reduced form of the proteinogenic amino acid l-alanine (Fig. 3). Given the evidence of ammonia in the interstellar medium, this reaction could have occurred on interstellar icy dust particles, and the resulting amino alcohols could have been subsequently embedded in comets and asteroids. During aqueous alteration of the parent body, an oxidation process might have allowed the formation of l-alanine. Thereby, the discovery of an enantiomeric excess of the *R*-(+)-enantiomer of a propylene oxide derivative (*53*) could be related to the reported *ee* of the l-enantiomer of some meteoritic amino acids (*67*, *68*).

Besides the substantial improvement of our CD and anisotropy spectra in terms of resolution and mirroring effect down to the far UV region, we believe that our data will stimulate future studies on establishing a unified view of the CPL scenario considering (i) the chiroptical properties of key biomolecules, (ii) the hypothesis that our Solar System encountered *l*-CPL radiation at the time of its formation, and (iii) the detection of chiral molecules in meteorites and samples returned from the asteroid sample-return missions Hayabusa2 and OSIRIS-REx.

## MATERIALS AND METHODS

### Chemicals

Enantiopure standards of *R*-(+)-propylene oxide (≥98.5%) and *S*-(−)-propylene oxide (not specified) were purchased from Sigma-Aldrich (Germany). Both standards were of high optical purity (99% *ee*) and were used without any further purification. *R*-(+)-camphor (98%) used for calibration purposes of the gas cell before recording CD spectra was purchased from Sigma-Aldrich (Germany).

### Experimental design

Gas-phase absorption spectra, electronic CD, and anisotropy spectra of individual enantiomers were recorded at the AU-CD beam line on ASTRID2 at Aarhus University (Denmark) (*69*, *70*). A temperature- and pressure-controlled gas cell has been built for this purpose and is described in detail elsewhere (*52*). The cell is made of stainless steel with an optical path length of 500 mm and an inner diameter of 18 mm. The vapor pressure of the samples was monitored by a type 631D Baratron heated manometer (MKS Instruments). CaF_2_ windows used at the extremities of the gas cell connected the apparatus with the photoelastic modulator to produce the CPL at one end and a photomultiplier tube detector at the other. Heating tapes and cartridge heaters allow heating of the entire gas cell up to 200°C, and we can monitor and locally control the temperature at nine different points of the gas cell assembly in case of low vapor pressure from a sample. This was needed for earlier measurements on amino acids (*2*), but the vapor pressure of propylene oxide was high enough to allow operation of the gas cell at room temperature. However, differential heating of the windows to higher temperatures compared to the main body of the gas cell avoided local condensation of analytes. Therefore, throughout all measurements, the temperature of the CaF_2_ windows was set to 30°C. Two all-metal valves are mounted on the main gas cell tube: one connects, via a flexible hose, to a Varian V70 turbo pump, while the other valve connects to the sample reservoir, a 25-ml single-neck round-bottom flask, connected at the center of the gas cell. This allows the pumping speed and sample inlet rate to the gas cell to be adjusted. Measured pressures increased rapidly above 0.8 mbar without sample flow, while it was kept in between 0.03 and 0.16 mbar for *R*-(+)- and *S*-(−)-propylene oxide during absorption and CD measurements.

### Gas-phase synchrotron radiation CD and anisotropy spectroscopy

The operation of the experimental set up was confirmed through measurement of *R*-(+)-camphor, for which the CD spectrum in the gas phase with a characteristic transition at about 300 nm is known (*71*). The absorption ε and CD spectra ∆ε of each propylene oxide enantiomer were recorded simultaneously in the gas phase. The gas cell was operated under flow conditions, where the valves to the sample and the turbo pump were partially open, to ensure a continuous renewal of the gas in the cell. This was done to avoid a build-up of any photodecomposition products. We were not able to maintain constant pressure under continuous flow conditions of each enantiomer but observed a steady increase of the pressure throughout the sample scans. Comparing different sample scans with each other, however, confirmed that the increase in pressure was due to the sample itself and not from other impurities such as water or decomposition products. The sample pressure was recorded for each data point in a spectrum, and all spectra are pressure-corrected.

The anisotropy spectra *g*(λ) = ∆ε/ε could be obtained as the absorbance of the gas, ε, was simultaneously measured with the CD. Absorbance is calculated using the regulated high voltage applied to photodetector and the gain value for detector, which has been determined through measurement on a series of samples on both the AU-CD beamline and a calibrated photospectrometer (Evolution 300, Thermo Scientific) (*72*). Approximately 10 ml of propylene oxide was added to a 25-ml pear-shaped glass container attached to the center of the gas cell by an all-metal valve. This amount of sample proved to be enough for multiple measurement runs. Before CD measurements, the sample was purged of dissolved gases by three freeze-pump-thaw cycles, and the valve was fully open to the cell to remove air or water vapor coming off the samples.

The gas-phase spectra were recorded from 4.4 to 9.5 eV (280 to 130 nm). Sample vapor pressures were chosen so that the optimal optical density near 0.8 providing the best signal-to-noise ratio was obtained. Multiple baseline scans of the empty gas cell were recorded before and after each propylene oxide enantiomer. Fortunately, the gas cell was designed to quickly be taken apart, cleaned, and reassembled as extensive white deposits within the cell and sample valve were observed. After extensive cleaning and remounting of the sample inlet for the gas cell, we pumped down the cell for several hours before running new baselines and the opposite enantiomer.

The CD spectra of propylene oxide enantiomers could be scaled directly with the cell pressure during measurements, and the absolute CD signal (corrected for gas density and path length) could be calculated. Thus, the CD spectra shown are in units of molar CD (per molar per centimeter) often referred to as Delta epsilon units. The CD spectra were mildly smoothed with a seven-point Savitzky-Golay filter.

### Computational details

The initial molecular structure of propylene oxide was built using GaussView (version 6). As the molecule exhibits no internal degrees of freedom, solely one conformer was considered. This structure was further optimized with Gaussian16, using a combination of different methods and different basis sets. We performed a single-point energy calculation with zero-point corrected energies including frequency calculations to verify the nature of true minima. Three methods previously applied for our amino acid gas-phase spectra were evaluated: CAM-B3LYP, ωB97X-D, and MO6-2X (*52*).

TD-DFT calculations were completed on the optimized structure using the three functionals combined with different basis sets. Two hundred excited states were calculated for the optimized conformer. Last, the combination of the range-separated functional CAM-B3LYP method with the quadruple-ζ with polarization and diffuse functions: aug-cc-pVQZ leads to the best fit between experiment and theory. To compare the theoretical data with the spectral shapes of the experiments, the theoretical electronic CD transitions have been convoluted by means of summing rotatory strength weighted Gaussian distribution functions with FWHM of 0.12 eV.

## Supplementary Materials

### This PDF file includes:

Supplementary Text Figs. S1 to S3 Tables S1 to S5 References

## References

[1] B. A. McGuire, P. B. Carroll, R. A. Loomis, I. A. Finneran, P. R. Jewell, A. J. Remijan, G. A. Blake,Discovery of the interstellar chiral molecule propylene oxide (CH_3_CHCH_2_O). Science 352,1449–1452 (2016). 27303055 10.1126/science.aae0328

[2] J. R. Brandt, F. Salerno, M. J. Fuchter,The added value of small-molecule chirality in technological applications. Nat. Rev. Chem. 1,1–12 (2017).

[3] F. Lancia, A. Ryabchun, N. Katsonis,Life-like motion driven by artificial molecular machines. Nat. Rev. Chem. 3,536–551 (2019).

[4] T. Leigh, P. Fernandez-Trillo,Helical polymers for biological and medical applications. Nat. Rev. Chem. 4,291–310 (2020). 10.1038/s41570-020-0180-5 37127955

[5] Z. Liu, H. Du, J. Li, L. Lu, Z.-Y. Li, N. X. Fang,Nano-kirigami with giant optical chirality. Sci. Adv. 4,eaat4436 (2018). 29984308 10.1126/sciadv.aat4436 PMC6035038

[6] G. Long, R. Sabatini, M. I. Saidaminov, G. Lakhwani, A. Rasmita, X. Liu, E. H. Sargent, W. Gao,Chiral-perovskite optoelectronics. Nat. Rev. Mater. 5,423–439 (2020).

[7] R. Naaman, Y. Paltiel, D. H. Waldeck,Chiral molecules and the electron spin. Nat. Rev. Chem. 3,250–260 (2019).

[8] G. Qu, A. Li, C. G. Acevedo-Rocha, Z. Sun, M. T. Reetz,The crucial role of methodology development in directed evolution of selective enzymes. Angew. Chem. Int. Ed. 59,13204–13231 (2020). 10.1002/anie.201901491 31267627

[9] G. F. Joyce, G. M. Visser, C. A. A. van Boeckel, J. H. van Boom, L. E. Orgel, J. van Westrenen,Chiral selection in poly(C)-directed synthesis of oligo(G). Nature 310,602–604 (1984). 6462250 10.1038/310602a0

[10] W. A. Bonner,The origin and amplification of biomolecular chirality. Orig. Life Evol. Biosph. 21,59–111 (1991). 1758688 10.1007/BF01809580

[11] F. C. Frank,On spontaneous asymmetric synthesis. Biochim. Biophys. Acta 11,459–463 (1953). 13105666 10.1016/0006-3002(53)90082-1

[12] M. Klussmann, T. Izumi, A. J. White, A. Armstrong, D. G. Blackmond,Emergence of solution-phase homochirality via crystal engineering of amino acids. J. Am. Chem. Soc. 129,7657–7660 (2007). 17530759 10.1021/ja0708870

[13] D. K. Kondepudi, R. J. Kaufman, N. Singh,Chiral symmetry breaking in sodium chlorate crystallization. Science 250,975–976 (1990). 17746924 10.1126/science.250.4983.975

[14] M. Quack,How important is parity violation for molecular and biomolecular chirality? Angew. Chem. Int. Ed. 41,4618–4630 (2002). 10.1002/anie.200290005 12481315

[15] J. B. Ribo, J. Crusats, F. Sagues, J. Claret, R. Rubires,Chiral sign induction by vortices during the formation of mesophases in stirred solutions. Science 292,2063–2066 (2001). 11408653 10.1126/science.1060835

[16] G. L. Rikken, E. Raupach,Enantioselective magnetochiral photochemistry. Nature 405,932–935 (2000). 10879530 10.1038/35016043

[17] R. A. Rosenberg, M. Abu Haija, P. J. Ryan,Chiral-selective chemistry induced by spin-polarized secondary electrons from a magnetic substrate. Phys. Rev. Lett. 101,178301 (2008). 18999792 10.1103/PhysRevLett.101.178301

[18] K. Soai, S. Osanai, K. Kadowaki, S. Yonekubo, T. Shibata, I. Sato,*d*- and *l*-Quartz-promoted highly enantioselective synthesis of a chiral organic compound. J. Am. Chem. Soc. 121,11235–11236 (1999).

[19] G. Tranter,Parity-violating energy differences of chiral minerals and the origin of biomolecular homochirality. Nature 318,172–173 (1985).

[20] F. Vester, T. Ulbricht, H. Krauch,Optische Aktivität und die Paritätsverletzung im β-Zerfall. Naturwissenschaften 46,68–68 (1959).

[21] P. De Marcellus, C. Meinert, M. Nuevo, J.-J. Filippi, G. Danger, D. Deboffle, L. Nahon, L. Le Sergeant d’Hendecourt, U. J. Meierhenrich,Non-racemic amino acid production by ultraviolet irradiation of achiral interstellar ice analogs with circularly polarized light. Astrophys. J. Lett. 727,L27 (2011).

[22] J. J. Flores, W. A. Bonner, G. A. Massey,Asymmetric photolysis of (RS)-leucine with circularly polarized ultraviolet light. J. Am. Chem. Soc. 99,3622–3625 (1977). 858868 10.1021/ja00453a018

[23] U. J. Meierhenrich, L. Nahon, C. Alcaraz, J. H. Bredehöft, S. V. Hoffmann, B. Barbier, A. Brack,Asymmetric vacuum UV photolysis of the amino acid leucine in the solid state. Angew. Chem. Int. Ed. 44,5630–5634 (2005). 10.1002/anie.200501311 16035020

[24] C. Meinert, S. V. Hoffmann, P. Cassam-Chenaï, A. C. Evans, C. Giri, L. Nahon, U. J. Meierhenrich,Photonenergy-controlled symmetry breaking with circularly polarized light. Angew. Chem. Int. Ed. 53,210–214 (2014). 10.1002/anie.201307855 24227543

[25] H. Nishino, A. Kosaka, G. A. Hembury, F. Aoki, K. Miyauchi, H. Shitomi, H. Onuki, Y. Inoue,Absolute asymmetric photoreactions of aliphatic amino acids by circularly polarized synchrotron radiation: Critically pH-dependent photo behavior. J. Am. Chem. Soc. 124,11618–11627 (2002). 12296726 10.1021/ja025959w

[26] W. L. Noorduin, A. A. C. Bode, M. van der Meijden, H. Meekes, A. F. van Etteger, W. J. P. van Enckevort, P. C. M. Christianen, B. Kaptein, R. M. Kellogg, T. Rasing, E. Vlieg,Complete chiral symmetry breaking of an amino acid derivative directed by circularly polarized light. Nat. Chem. 1,729–732 (2009). 21124360 10.1038/nchem.416

[27] B. Norden,Was photoresolution of amino acids the origin of optical activity in life? Nature 266,567–568 (1977). 859626 10.1038/266567a0

[28] J. A. L. Bel,Sur les relations qui existent entre les formules atomiques des corps organiques et le pouvoir rotatoire de leurs dissolutions. Bull. Soc. Chim. Paris 22,337–347 (1874).

[29] N. P. Huck, W. F. Jager, B. De Lange, B. L. Feringa,Dynamic control and amplification of molecular chirality by circular polarized light. Science 273,1686–1688 (1996).

[30] J.-Y. Kim, J. Yeom, H. Calcaterra, G. Zhao, P. Zhang, N. Kotov,Assembly of gold nanoparticles into chiral superstructures driven by circularly polarized light. J. Am. Chem. Soc. 141,11739–11744 (2019). 31329438 10.1021/jacs.9b00700 PMC7263784

[31] A. D. Garcia, C. Meinert, H. Sugahara, N. C. Jones, S. V. Hoffmann, U. J. Meierhenrich,The astrophysical formation of asymmetric molecules and the emergence of a chiral bias. Life 9,29 (2019). 30884807 10.3390/life9010029 PMC6463258

[32] I. Myrgorodska, C. Meinert, Z. Martins, L. Le Sergeant d’Hendecourt, U. J. Meierhenrich,Molecular chirality in meteorites and interstellar ices, and the chirality experiment on board the ESA cometary Rosetta mission. Angew. Chem. Int. Ed. 54,1402–1412 (2015). 10.1002/anie.201409354 25431250

[33] H. K. Bisoyi, Q. Li,Light-directed dynamic chirality inversion in functional self-organized helical superstructures. Angew. Chem. Int. Ed. 55,2994–3010 (2016). 10.1002/anie.201505520 26764018

[34] C. He, G. Yang, Y. Kuai, S. Shan, L. Yang, J. Hu, D. Zhang, Q. Zhang, G. Zou,Dissymmetry enhancement in enantioselective synthesis of helical polydiacetylene by application of superchiral light. Nat. Commun. 9,5117 (2018). 30504770 10.1038/s41467-018-07533-y PMC6269450

[35] H. Kagan, A. Moradpour, J. F. Nicoud, G. Balavoine, G. Tsoucaris,Photochemistry with circularly polarized light. Synthesis of optically active hexahelicene. J. Am. Chem. Soc. 93,2353–2354 (2002).

[36] S. T. Wu, Z. W. Cai, Q. Y. Ye, C. H. Weng, X. H. Huang, X. L. Hu, C. C. Huang, N. F. Zhuang,Enantioselective synthesis of a chiral coordination polymer with circularly polarized visible laser. Angew. Chem. Int. Ed. 53,12860–12864 (2014). 10.1002/anie.201407026 25251289

[37] J. Lu, Y. Xue, K. Bernardino, N. N. Zhang, W. R. Gomes, N. S. Ramesar, S. Liu, Z. Hu, T. Sun, A. F. de Moura, N. A. Kotov, K. Liu,Enhanced optical asymmetry in supramolecular chiroplasmonic assemblies with long-range order. Science 371,1368–1374 (2021). 33632891 10.1126/science.abd8576

[38] L. Wan, J. Wade, F. Salerno, O. Arteaga, B. Laidlaw, X. Wang, T. Penfold, M. J. Fuchter, A. J. Campbell,Inverting the handedness of circularly polarized luminescence from light-emitting polymers using film thickness. ACS Nano 13,8099–8105 (2019). 31241299 10.1021/acsnano.9b02940

[39] M. D. Ward, J. Wade, X. Shi, J. Nelson, A. J. Campbell, M. J. Fuchter,Highly selective high-speed circularly polarized photodiodes based on π-conjugated polymers. Adv. Opt. Mater. 10,2101044 (2021).

[40] G. Pescitelli, L. Di Bari, N. Berova,Conformational aspects in the studies of organic compounds by electronic circular dichroism. Chem. Soc. Rev. 40,4603–4625 (2011). 21677932 10.1039/c1cs15036g

[41] W. Kuhn, E. Braun,Photochemische Erzeugung optisch aktiver Stoffe. Naturwissenschaften 17,227–228 (1929).

[42] W. Kuhn, E. Knopf,Photochemische Erzeugung optisch aktiver Stoffe. Naturwissenschaften 18,183 (1930).

[43] C. Meinert, J. H. Bredehöft, J. J. Filippi, Y. Baraud, L. Nahon, F. Wien, N. C. Jones, S. V. Hoffmann, U. J. Meierhenrich,Anisotropy spectra of amino acids. Angew. Chem. Int. Ed. 51,4484–4487 (2012). 10.1002/anie.201108997 22438137

[44] A. Breest, P. Ochmann, F. Pulm, K. H. Gödderz, M. Carnell, J. Hormes,Experimental circular dichroism and VUV spectra of substituted oxiranes and thiiranes. Mol. Phys. 82,539–551 (1994).

[45] M. Carnell,Experimental and quantum-theoretical investigation of the circular dichroism spectrum of R-methyloxirane. Chem. Phys. Lett. 180,477–481 (1991).

[46] D. Cohen, M. Levi, H. Basch, A. Gedanken,Excited electronic states of optically active substituted ethylene oxides:(−)-(S)-2-methyloxirane and (−)-(S, S)-2, 3-dimethyloxirane. J. Am. Chem. Soc. 105,1738–1742 (1983).

[47] J. Cukras, J. Kauczor, P. Norman, A. Rizzo, G. L. J. A. Rikken, S. Coriani,A complex-polarization-propagator protocol for magneto-chiral axial dichroism and birefringence dispersion. Phys. Chem. Chem. Phys. 18,13267–13279 (2016). 27118603 10.1039/c6cp01465h

[48] M. M. Rafiee Fanood, I. Powis, M. H. Janssen,Chiral asymmetry in the multiphoton ionization of methyloxirane using femtosecond electron–ion coincidence imaging. J. Phys. Chem. A 118,11541–11546 (2014). 25402546 10.1021/jp5113125

[49] L. Alagna, S. di Fonzo, T. Prosperi, S. Turchini, P. Lazzeretti, M. Malagoli, R. Zanasi, C. R. Natoli, P. J. Stephens,Random phase approximation calculations of K-edge rotational strengths of chiral molecules: Propylene oxide. Chem. Phys. Lett. 223,402–410 (1994).

[50] M. Hodecker, M. Biczysko, A. Dreuw, V. Barone,Simulation of vacuum UV absorption and electronic circular dichroism spectra of methyl oxirane: The role of vibrational effects. J. Chem. Theory Comput. 12,2820–2833 (2016). 27159495 10.1021/acs.jctc.6b00121 PMC5612404

[51] D. Kroner,Laser-driven electron dynamics for circular dichroism in mass spectrometry: From one-photon excitations to multiphoton ionization. Phys. Chem. Chem. Phys. 17,19643–19655 (2015). 26151731 10.1039/c5cp02193f

[52] C. Meinert, A. D. Garcia, J. Topin, N. C. Jones, M. Diekmann, R. Berger, L. Nahon, S. V. Hoffmann, U. J. Meierhenrich,Amino acid gas phase circular dichroism and implications for the origin of biomolecular asymmetry. Nat. Commun. 13,502 (2022). 35082305 10.1038/s41467-022-28184-0 PMC8792022

[53] S. Pizzarello, C. T. Yarnes,Chiral molecules in space and their possible passage to planetary bodies recorded by meteorites. Earth Planet. Sci. Lett. 496,198–205 (2018).

[54] T. Yanai, D. P. Tew, N. C. Handy,A new hybrid exchange–correlation functional using the Coulomb-attenuating method (CAM-B3LYP). Chem. Phys. Lett. 393,51–57 (2004).

[55] T. Miyahara, J.-y. Hasegawa, H. Nakatsuji,Circular dichroism and absorption spectroscopy for three-membered ring compounds using Symmetry-Adapted Cluster-Configuration Interaction (SAC-CI) method. Bull. Chem. Soc. Jpn. 82,1215–1226 (2009).

[56] J. Neugebauer, E. J. Baerends, M. Nooijen, J. Autschbach,Importance of vibronic effects on the circular dichroism spectrum of dimethyloxirane. J. Chem. Phys. 122,234305 (2005). 16008439 10.1063/1.1927519

[57] J. R. Cronin, S. Pizzarello,Enantiomeric excesses in meteoritic amino acids. Science 275,951–955 (1997). 9020072 10.1126/science.275.5302.951

[58] D. P. Glavin, J. P. Dworkin,Enrichment of the amino acid l-isovaline by aqueous alteration on CI and CM meteorite parent bodies. Proc. Natl. Acad. Sci. U.S.A. 106,5487–5492 (2009). 19289826 10.1073/pnas.0811618106 PMC2667035

[59] G. Cooper, A. C. Rios,Enantiomer excesses of rare and common sugar derivatives in carbonaceous meteorites. Proc. Natl. Acad. Sci. U.S.A. 113,E3322–E3331 (2016). 27247410 10.1073/pnas.1603030113 PMC4914185

[60] J. Bailey, A. Chrysostomou, J. H. Hough, T. M. Gledhill, A. McCall, S. Clark, F. Ménard, M. Tamura,Circular polarization in star- formation regions: Implications for biomolecular homochirality. Science 281,672–674 (1998). 9685254

[61] M. Buschermohle, D. C. B. Whittet, A. Chrysostomou, J. H. Hough, P. W. Lucas, A. J. Adamson, B. A. Whitney, M. J. Wolff,An extended search for circularly polarized infrared radiation from the OMC-1 region of Orion. Astrophys. J. 624,821–826 (2005).

[62] A. Chrysostomou, P. W. Lucas, J. H. Hough,Circular polarimetry reveals helical magnetic fields in the young stellar object HH 135-136. Nature 450,71–73 (2007). 17972878 10.1038/nature06220

[63] J. Kwon, M. Tamura, P. W. Lucas, J. Hashimoto, N. Kusakabe, R. Kandori, Y. Nakajima, T. Nagayama, T. Nagata, J. H. Hough,Near-infrared circular polarization images of NGC 6334-V. Astrophys. J. 765,L6 (2013).

[64] A. Bergantini, M. J. Abplanalp, P. Pokhilko, A. I. Krylov, C. N. Shingledecker, E. Herbst, R. I. Kaiser,A combined experimental and theoretical study on the formation of interstellar propylene oxide (CH_3_CHCH_2_O)—A chiral molecule. Astrophys. J. 860,108 (2018).

[65] D. R. Paulson, A. S. Murray, D. Bennett, E. Mills Jr., V. O. Terry, S. D. Lopez,Photochemistry of epoxides. 3. Direct irradiation of propylene oxide in the gas phase. J. Org. Chem. 42,1252–1254 (1977).

[66] J. C. Forbes, J. Alves, D. N. C. Lin,A solar system formation analogue in the Ophiuchus star-forming complex. Nat. Astron. 5,1009–1016 (2021).

[67] J. R. Cronin, S. Pizzarello,Amino acid enantiomer excesses in meteorites: Origin and significance. Adv. Space Res. 23,293–299 (1999).

[68] S. Pizzarello, J. R. Cronin,Non-racemic amino acids in the Murray and Murchison meteorites. Geochim. Cosmochim. Acta 64,329–338 (2000). 11543420 10.1016/s0016-7037(99)00280-x

[69] A. J. Miles, S. V. Hoffmann, Y. Tao, R. W. Janes, B. A. Wallace,Synchrotron radiation circular dichroism (SRCD) spectroscopy: New beamlines and new applications in biology. Spectroscopy 21,245–255 (2007).

[70] A. J. Miles, R. W. Janes, A. Brown, D. T. Clarke, J. C. Sutherland, Y. Tao, B. A. Wallace, S. V. Hoffmann,Light flux density threshold at which protein denaturation is induced by synchrotron radiation circular dichroism beamlines. J. Synchrotron Radiat. 15,420–422 (2008). 18552437 10.1107/S0909049508009606

[71] F. Pulm, J. Schramm, J. Hormes, S. Grimme, S. D. Peyerimhoff,Theoretical and experimental investigations of the electronic circular dichroism and absorption spectra of bicyclic ketones. Chem. Phys. 224,143–155 (1997).

[72] A. C. Evans, C. Meinert, J. H. Bredehoeft, C. Giri, N. C. Jones, S. V. Hoffmann, U. J. Meierhenrich,Anisotropy spectra for enantiomeric differentiation of biomolecular building blocks. Top. Curr. Chem. 341,271–299 (2013). 23839281 10.1007/128_2013_442

[73] J. Bloino, M. Biczysko, F. Santoro, V. Barone,General approach to compute vibrationally resolved one-photon electronic spectra. J. Chem. Theory Comput. 6,1256–1274 (2010). 10.1021/ct8004744 26610221

[74] A. Rizzo, O. Vahtras,*Ab initio* study of excited state electronic circular dichroism. Two prototype cases: Methyl oxirane and R-(+)-1,1′-bi(2-naphthol). J. Chem. Phys. 134,244109 (2011). 21721614 10.1063/1.3602219

