# Supplementary material for: Chiroptical activity of gas-phase propylene oxide predicting the handedness of interstellar circular polarization in the presolar nebula
Source: Sci Adv. 2022 Nov 18;8(46):eadd4614. doi: 10.1126/sciadv.add4614 (PMC9674286; doi:10.1126/sciadv.add4614)
Supplement: Supplementary file 1 — Supplementary Text Figs. S1 to S3 Tables S1 to S5 References [file sciadv.add4614_sm.pdf]

Science Advances
AAAS

Supplementary Materials for

Chiroptical activity of gas-phase propylene oxide predicting the handedness of interstellar circular polarization in the presolar nebula

Adrien D. Garcia *et al.*

Corresponding author: Cornelia Meinert, cornelia.meinert@univ-cotedazur.fr

Sci. Adv. **8**, eadd4614 (2022)
DOI: 10.1126/sciadv.add4614

This PDF file includes:

Supplementary Text
Figs. S1 to S3
Tables S1 to S5
References

Supplementary Text

Gas phase absorption, circular dichroism and anisotropy spectra of propylene oxide

To provide the best representative absorbance data of both propylene oxide enantiomers, spectra were measured at various gas pressures. Individually recorded absorbance spectra could be normalized based on the vapour pressure to yield spectra of similar shape and magnitude. The absorbance spectra of both the *R*-(+) and the *S*-(-)-enantiomer of propylene oxide are shown in Fig. S2 with the corresponding relative absorption values for the most prominent gas phase electronic excitations summarized in Table S1. Given that our experimental absorbance and CD spectra discern different levels of vibrational fine structure, the labelling of the states in Table S1 and Table S2 differ to avoid misleading cross-linking.

Another important parameter related to chiroptical activity is the anisotropy factor g. It is useful to quantitatively describe the chemical kinetics of enantioselective photoreactions, as it is directly linked to the inequality of two enantiomers towards the helicities of CPL (*42*). It is expressed as the difference in molar extinction coefficient between *r*- and *l*-CPL ($\Delta\varepsilon$) divided by the global molar extinction coefficient (ε):

$$g = \frac{\varepsilon_{(l-\mathrm{CPL})} - \varepsilon_{(r-\mathrm{CPL})}}{\varepsilon} = \frac{\Delta\varepsilon}{\varepsilon} = \frac{\Delta\varepsilon}{(\varepsilon_{(l-\mathrm{CPL})} + \varepsilon_{(r-\mathrm{CPL})})/2}$$

The anisotropy factor g offers the possibility to evaluate the net effect of CPL in asymmetric photoreactions relating it to the inducible enantiomeric excess (*ee*). The minimum inducible *ee* reported in Figure 2 and Table S3 was calculated using the anisotropy factor g and the extent of the photolysis rate ξ by the following inequation (*43*):

$$|\%ee| \geq (1 - (1 - \xi)^{\frac{|g|}{2}}) \times 100$$

The sign of *ee* is then determined based on the sign of g at a particular wavelength and helicity of CPL. Anisotropy spectroscopy provides therefore direct information on the polarization- and wavelength-dependent molecular anisotropy g inherent to CPL-induced photochemical processes, as well as the potential outcome in terms of inducible optical purity (*ee*).

Numerical integration over all g bands in the measured UV wavelength range

Numerical integration was performed in Matlab using a so-called *trapezoidal method*. Specifically, the integration over each g band was approximated by breaking the corresponding areas highlighted in blue in Fig. S3 into trapezoids with spacings defined by the steps taken in the anisotropy spectroscopy experiments, i.e. 0.25 nm in the wavelength range 130–181 nm and 1 nm in the range 181–280 nm with the exception of the first and last trapezoids of each band due to zero crossings. The wavelengths corresponding to the zero crossings – i.e. limits of integration – were established as intersections between the line segments connecting two consecutive data points with opposite %*ee* signs and the x-axis.

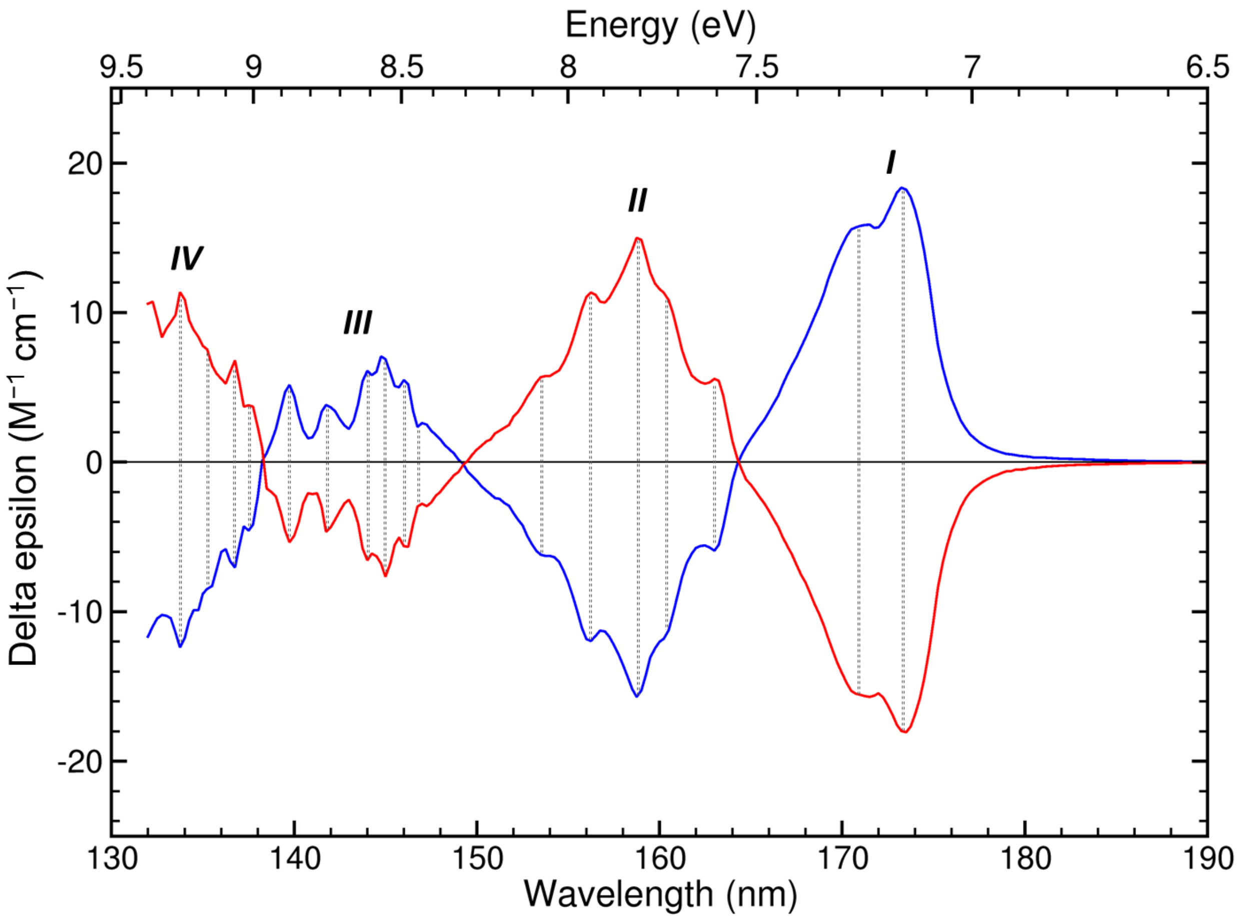

Fig. S1. Gas phase circular dichroism spectra of propylene oxide in the vacuum UV spectral range. Experimental gas phase CD spectra of *R*-(+)-propylene oxide (red) and *S*-(-)-propylene oxide (blue) recorded at 297 K using a 500 mm cell on the AU-CD beam line. Dotted lines indicate the 17 most dominant resolved electronic and vibronic transitions.

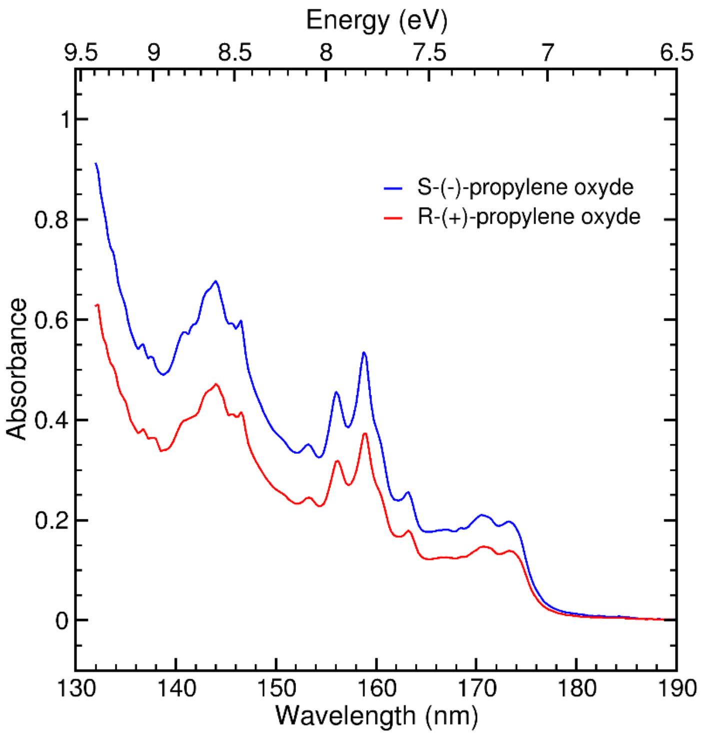

Fig. S2. High resolution gas phase absorbance spectra of propylene oxide in the vacuum UV spectral range. Gas phase absorbance spectra of *R*-(+)-propylene oxide (red) and *S*-(-)-propylene oxide (blue) recorded at 297 K using a 500 mm cell on the AU-CD beam line.

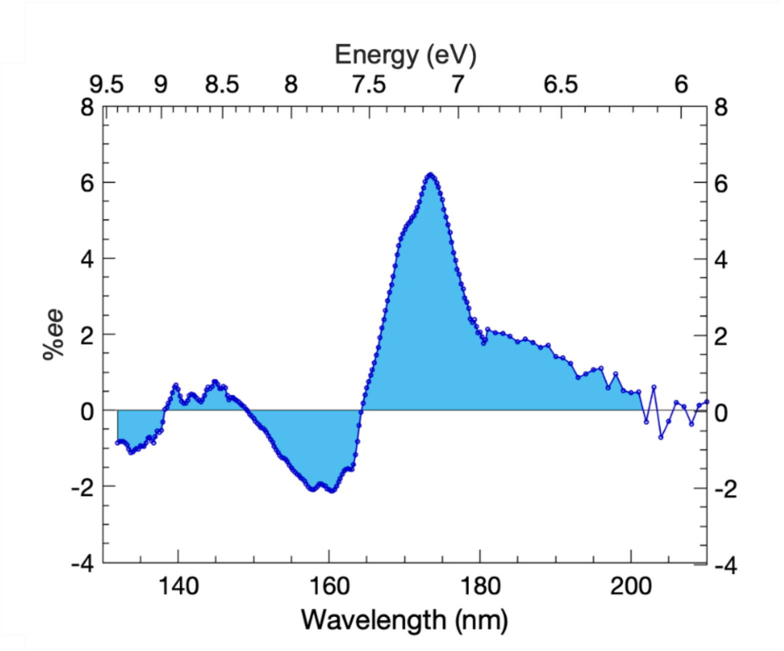

Fig. S3. Integrated area (blue) under the %*ee*-curve obtained for *S*-(-)-propylene oxide (dark blue) with an extent of reaction $\xi = 0.9999$.

Table S1.

Experimental electronic excitation energies (ΔE) and corresponding relative absorption (A) values[#] of the most prominent gas phase electronic transitions of (*R*)- and (*S*)-propylene oxide.

CD band	State	Exp. *R*-(+)-propylene oxide		Exp. *S*-(-)-propylene oxide	
		ΔE (eV)	A (a.u.)	ΔE (eV)	A (a.u.)
I	$^1S_{i}$	7.16	0.20	7.16	0.14
	$^1S_{ii}$	7.27	0.21	7.26	0.15
	$^1S_{iii}$	7.36	0.18	7.36	0.13
II	$^1S_{iv}$	7.59	0.26	7.59	0.18
	$^1S_{v}$	7.74	0.37	7.74	0.26
	$^1S_{vi}$	7.81	0.54	7.80	0.37
	$^1S_{vii}$	7.95	0.46	7.93	0.32
	$^1S_{viii}$	8.09	0.35	8.09	0.25
	$^1S_{ix}$	8.22	0.36	8.22	0.25
III	$^1S_{x}$	8.46	0.60	8.46	0.42
	$^1S_{xi}$	8.52	0.59	8.52	0.41
	$^1S_{xii}$	8.61	0.68	8.61	0.47
	$^1S_{xiii}$	8.67	0.65	8.67	0.45
	$^1S_{xiv}$	8.81	0.57	8.81	0.40
IV	$^1S_{xv}$	9.02	0.53	9.00	0.36
	$^1S_{xvi}$	9.07	0.55	9.07	0.38
	$^1S_{xvii}$	9.18	0.62	9.18	0.44
	$^1S_{xviii}$	9.27	0.73	9.27	0.51

[#]Only absorption bands that are present in the absorption spectra of both enantiomers are included.

Table S2.

Experimental electronic excitation energies (Δ*E*) and electronic circular dichroism (Δ*ε*) of the most prominent gas phase dichroic transitions of (*R*)- and (*S*)-propylene oxide (PO). Comparison with previously reported experimental results.

		Present study				Cohen *et al.* 1983 (*46*)		Carnell *et al.* 1991 (*45*)		Breest *et al.* 1994 (*44*)	
		Exp. (*R*)-PO		Exp. (*S*)-PO		Exp. (*S*)-PO		Exp. (*R*)-PO		Exp. (*R*)-PO	
CD	state	Δ*E* (eV)	Δ*ε* (M^{-1}×cm^{-1})	Δ*E* (eV)	Δ*ε* (M^{-1}×cm^{-1})	Δ*E* (eV)	Δ*ε* (M^{-1}×cm^{-1})	Δ*E* (eV)	Δ*ε* (M^{-1}×cm^{-1})	Δ*E* (eV)	Δ*ε* (M^{-1}×cm^{-1})
**I**	$^1S_1$	7.15	−18.06	7.16	18.36	7.12	11.80	7.07	−12.56	7.08	−12.50
	$^1S_2$	7.23	−15.69	7.23	15.87						
**II**	$^1S_3$	7.61	5.58	7.61	−5.93						
	$^1S_4$	7.72	10.84	7.72	−11.24						
	$^1S_5$	7.81	14.98	7.81	−15.70	7.75	−10.80	7.70	6.98	7.70	5.90
	$^1S_6$	7.94	11.34	7.94	−11.97					7.85	-
	$^1S_7$	8.08	5.68	8.08	−6.20						
**III**	$^1S_8$	8.42	−2.93	8.43	2.62						
	$^1S_9$	8.48	−5.66	8.49	5.49						
	$^1S_{10}$	8.55	−7.67	8.57	7.07			8.50¶	−4.00	8.35¶	−4.10
	$^1S_{11}$	8.61	−6.57	8.61	6.12						
	$^1S_{12}$	8.75	−4.66	8.75	3.83						
	$^1S_{13}$	8.87	−5.36	8.87	5.16						
**IV**	$^1S_{14}$	9.02	3.81	9.02	−4.58						
	$^1S_{15}$	9.07	6.81	9.07	−7.05						
	$^1S_{16}$	9.17	7.53	9.15	−8.29						
	$^1S_{17}$	9.27	11.38	9.27	−12.38						

¶No distinct assignment of the previously reported higher-lying excited electronic state with our experimental results possible due to only weak and inconsistent features in this energy range.

Table S3.

Anisotropy factor g and the lower limit of inducible enantiomeric excess of the *R*-(+)-enantiomer (ee_R) by asymmetric photolysis of racemic gas-phase propylene oxide (PO) with *l*-CPL at the extent of reaction $\xi = 0.9999$ at energies corresponding to the extrema of anisotropy bands I–IV calculated based on the equation provided by Meinert *et al*. 2012 (*43*).

CD band	Exp. *R*-(+)-PO				Exp. *S*-(-)-PO			
	ΔE (eV)	λ (nm)	g ($\times 10^{-2}$)	ee_R (%)	ΔE (eV)	λ (nm)	g ($\times 10^{-2}$)	ee_R (%)
**I**	7.15	173.5	−1.33	5.92	7.15	173.5	1.35	6.02
**II**	7.72	160.5	0.44	-2.01	7.72	160.5	−0.46	−2.11
**III**	8.55	145.0	−0.18	0.84	8.55	145.0	0.17	0.76
**IV**	9.27	133.8	0.23	-1.05	9.27	133.8	−0.24	−1.12

Table S4a.

Comparison of our TD-DFT/CAM-B3LYP calculated vertical excitation energies (VE) and corresponding rotatory strengths (*R*) of *S*-(-)-propylene oxide computed with different basis sets.

CD band	TD-DFT/CAM-B3LYP 6-31+G*		6-311++G*		aug-cc-pVQZ		Def2QZVPP	
	VE (eV)	*R* (10^{-40} cgs)	VE (eV)	*R* (10^{-40} cgs)	VE (eV)	*R* (10^{-40} cgs)	VE (eV)	*R* (10^{-40} cgs)
**I**	7.14	19.32	7.19	19.68	7.16	15.88	7.91	33.87
	7.50	3.44	7.55	3.64	7.49	7.04	8.05	−3.76
**II**	7.60	−9.22	7.65	−8.67	7.58	−8.69	8.34	−2.12
	7.76	−9.15	7.81	−8.96	7.74	−7.33	8.55	−27.60
	7.87	−11.64	7.92	−11.21	7.88	−10.64	8.56	−0.66
**III**	8.36	−0.15	8.40	−0.36	8.32	−0.15	8.83	−11.11
	8.43	20.95	8.46	16.95	8.36	18.61	9.03	−7.83
	8.49	−7.84	8.52	−7.26	8.42	−9.91	9.11	21.32
	8.52	8.26	8.56	9.01	8.46	9.31	9.24	23.91
	8.68	7.87	8.72	7.57	8.57	−0.02	9.53	3.62
	8.75	−4.28	8.82	−3.91	8.60	2.65	9.85	−19.04
	8.92	−1.79	8.96	−2.96	8.67	−2.34	10.03	−49.01
**IV**	9.32	−5.35	9.36	−4.88	9.04	1.80	10.11	35.23
	9.46	−9.76	9.51	−6.35	9.18	5.07	10.23	−6.35
	9.58	−30.74	9.55	−20.80	9.22	−6.54	10.34	15.80
	9.61	−9.78	9.63	−16.55	9.29	−13.36	10.43	−6.27

Table S4b.

Comparison of our TD-DFT/MO6-2X calculated vertical excitation energies (VE) and corresponding rotatory strengths (*R*) of *S*-(-)-propylene oxide computed with different basis sets.

	TD-DFT/MO6-2X							
CD band	6-31+G*		6-311++G*		aug-cc-pVQZ		Def2QZVPP	
	VE (eV)	*R* (10^{-40} cgs)	VE (eV)	*R* (10^{-40} cgs)	VE (eV)	*R* (10^{-40} cgs)	VE (eV)	*R* (10^{-40} cgs)
**I**	7.37	22.76	7.43	21.40	7.44	18.25	8.19	9.76
	7.63	4.26	7.69	7.79	7.68	10.05	8.33	22.32
**II**	7.73	−9.77	7.75	−14.90	7.76	−13.56	8.57	−9.90
	7.83	−16.82	7.89	−13.36	7.86	−14.16	8.68	13.67
	7.95	−8.78	8.01	−8.42	7.99	−6.83	8.90	−28.69
**III**	8.19	−0.25	8.20	−0.56	8.17	−0.76	8.95	−16.89
	8.28	23.39	8.31	22.36	8.24	20.13	9.00	−0.09
	8.34	8.41	8.36	6.95	8.33	6.55	9.10	18.97
	8.53	−10.31	8.57	−9.45	8.56	−8.57	9.22	21.02
	8.79	4.44	8.86	3.73	8.75	1.63	9.79	−3.94
	8.85	−0.64	8.91	−0.99	8.78	4.66	9.99	−27.58
	9.05	−4.11	9.10	−7.67	8.88	−2.04	10.15	−26.31
**IV**	9.12	0.89	9.15	5.00	9.03	−0.61	10.22	7.77
	9.25	−9.24	9.28	−8.31	9.14	−7.73	10.35	−3.59
	9.44	−6.22	9.45	−4.20	9.20	−6.36	10.41	−8.08
	9.55	−4.18	9.54	10.51	9.22	1.80	10.44	21.24

Table S4c.

Comparison of our TD-DFT/ωB97X-D calculated vertical excitation energies (VE) and corresponding rotatory strengths (*R*) of *S*-(-)-propylene oxide computed with different basis sets.

CD band	TD-DFT/ωB97X-D 6-31+G*		6-311++G*		aug-cc-pVQZ		Def2QZVPP	
	VE (eV)	*R* (10^{-40} cgs)	VE (eV)	*R* (10^{-40} cgs)	VE (eV)	*R* (10^{-40} cgs)	VE (eV)	*R* (10^{-40} cgs)
**I**	7.31	17.64	7.34	18.01	7.25	12.24	8.18	35.51
	7.70	4.28	7.74	4.68	7.62	9.83	8.25	−4.78
**II**	7.75	−8.43	7.79	−8.10	7.64	−8.20	8.58	0.02
	7.94	−8.37	7.97	−8.10	7.82	6.20	8.836	−17.67
	8.05	−11.30	8.08	−11.25	7.99	−10.39	8.841	−5.06
**III**	8.53	1.00	8.56	1.10	8.41	−0.81	8.99	−23.64
	8.58	1.04	8.59	−2.06	8.45	15.35	9.30	−3.23
	8.64	7.40	8.66	9.21	8.48	−4.01	9.37	19.41
	8.70	11.90	8.72	10.90	8.55	9.37	9.54	29.16
	8.82	7.79	8.85	7.58	8.59	−4.80	9.80	2.82
	8.95	−6.78	8.99	−6.69	8.64	−0.15	10.12	−27.26
	9.07	−1.55	9.10	−3.01	8.70	1.69	10.24	−46.30
**IV**	9.48	−6.79	9.50	−6.45	9.10	1.06	10.38	42.18
	9.63	−10.07	9.65	−8.10	9.18	2.46	10.49	2.26
	9.74	−27.92	9.73	−21.51	9.23	−7.36	10.56	2.30
	9.78	−9.91	9.78	−12.19	9.32	−11.36	10.70	−10.44

Table S5a.

Comparison of our TD-DFT calculated vertical excitation energies (VE) and corresponding rotatory strengths (*R*) of propylene oxide (PO) with the previous literature. Only the studies where at least 5 excitation energies were calculated are cited and where available the transition assignments are stated.

Present study		Carnell *et al*. 1991 (*45*)				Miyahara *et al*. 2009 (*55*)				Bloino *et al*. 2010 (*73*)[$]			
TD-DFT/CAM-B3LYP (aug-cc-pvQZ), *R*-PO		MRDI-CI, *R*-PO				SAC-CI, *R*-PO				TD-DFT/CAM-B3LYP (aug-cc-pVTZ), *R*-PO		TD-DFT/B3LYP (aug-cc-pVTZ), *R*-PO	
VE (eV)	*R* (10^{-40} cgs)	Nature of excitation[¶]	VE (eV)	R_{length} (10^{-40} cgs)	R_{length} (10^{-40} cgs)	Nature of excitation	VE (eV)	R_{length} (10^{-40} cgs)	$R_{velocity}$ (10^{-40} cgs)	VE (eV)	R_{length} (10^{-40} cgs)	VE (eV)	R_{length} (10^{-40} cgs)
7.16	−15.88	$n_O \rightarrow R$(3s)	6.25/7.12[#]	−6.43	−5.39	$n_O \rightarrow R$(3s)	7.01	−10.492	−11.446	7.15	−16.63	6.56	−19.37
7.49	−7.04					$n_O \rightarrow R$(3p)	7.49	-3.950	−4.925	7.49	−6.81 (9.73)	6.99	13.01 (16.43)
7.58	8.69	$n_O \rightarrow R$(3p)	6.85	7.85	7.19	$n_O \rightarrow R$(3p)	7.56	4.865	4.787	7.58	8.72	7.03	0.88
7.74	7.33	$n_O \rightarrow R$(3p)	6.94	−4.97	−4.51	$n_O \rightarrow R$(3p)	7.71	5.463	6.117	7.74	7.81	7.11	2.55
7.88	10.64	$n_O \rightarrow R$(3p)	7.07	4.45	3.41	$\sigma \rightarrow R$(3s)	7.87	6.716	7.653	7.86	11.03	7.36	8.80
						$n_O \rightarrow R$(3d)	8.29	0.619	0.672				
8.32	0.15					$n_O \rightarrow R$(3d)	8.34	1.721	1.771	8.30	0.86	7.76	2.74
8.36	−18.61	$n_O \rightarrow R$(3d)	8.39	−1.84	−3.70	$n_O \rightarrow R$(3d)	8.36	−3.624	−3.794	8.36	−19.83	7.82	−3.50
8.42	9.91					$n_O \rightarrow R$(3d)	8.42	−2.867	−3.647				
8.46	−9.31					$\sigma \rightarrow R$(3p)	8.44	−10.302	−9.662				
8.57	0.02					$\sigma \rightarrow R$(3p)	8.46	−2.006	−2.468				
8.60	−2.65					$n_O \rightarrow R$(3d)	8.50	−1.319	−1.336				
8.67	2.34					$n_O \rightarrow R$(3d)	8.51	−0.137	0.169				
9.04	1.80					$\sigma \rightarrow R$(3p)	8.53	0.806	0.520				
						$n_O \rightarrow R$(4s)	8.65	1.431	1.357				
9.18	−5.07												
9.22	6.54												
9.29	13.36												
9.38	−3.00												

[¶]Reported by Breest *et al*. (*44*).

[#]Value if both the upper and lower states are described in their own set of molecular orbitals in MRD-CI expansion.

[$]Calculations for the basis sets: aug-cc-pV5Z, aug-cc-pVQZ, N07Tdiff, N07T, N07Ddiff and N07D are not reported for brevity.

Table S5b.

Comparison of our TD-DFT calculated vertical excitation energies (VE) and corresponding rotatory strengths (*R*) of propylene oxide (PO) with the previous literature. Only the studies where at least 5 excitation energies were calculated are cited and where available the transition assignments are stated.

**Present study**		**Rizzo *et al*. 2011 (*74*)**						**Kröner 2015 (*51*)**			**Hodecker *et al*. 2016 (*50*)[$]**			
**TD-DFT/CAM-B3LYP (aug-cc-pvQZ), *R*-PO**		**DFT/CAM-B3LYP (d-aug-cc-pVQZ), *R*-PO**		**DFT/B3LYP (d-aug-cc-pVQZ), *R*-PO**		**HF-SCF (d-aug-cc-pVQZ), *R*-PO**		**TD-CIS(D) (aug-cc-pVTZ), *R*-PO**			**EOM-CCSD (aug-cc-pVQZ), *R*-PO**		**TD-DFT/CAM-B3LYP (aug-cc-pVQZ), *R*-PO**	
**VE (eV)**	***R*** **(10^{-40} cgs)**	**VE (eV)**	***R*** **(10^{-3} a.u.)**	**VE (eV)**	***R*** **(10^{-3} a.u.)**	**VE (eV)**	***R*** **(10^{-3} a.u.)**	**Nature of excitation**	**VE (eV)**	***R*** **(10^{-40} cgs)**	**VE[a] (eV)**	**R_{length} (10^{-40} cgs)**	**VE[a] (eV)**	**R_{length} (10^{-40} cgs)**
7.16	−15.88	7.133	−41.260	6.483	−39.950	8.483	−14.940	$n_O \rightarrow R$(3s)	6.95	−17.000	7.43	−17.35	7.16	−15.89
7.49	−7.04	7.339	12.880	6.901	4.507	8.744	38.730	$n_O \rightarrow R$(3s)	7.01	12.300	7.68	−4.81	7.48	-7.15
7.58	8.69	7.592	23.590	6.930	10.480	9.060	2.543	$n_O \rightarrow R$(3sp)	7.10	4.160	7.88	9.12	7.58	8.79
7.74	7.33	7.702	9.181	6.965	25.930	9.218	−43.860	$n_O \rightarrow R$(3p)	7.29	7.370	8.09	6.86	7.74	7.32
7.88	10.64	7.848	18.140	7.266	18.540	9.305	−34.880	$\sigma \rightarrow R$(3s)	7.72	−0.521	8.25	10.46	7.87	10.54
8.32	0.15	8.149	−42.750	7.410	0.461	9.490	−36.900	$\sigma \rightarrow R$(3sp)	8.01	−16.300	8.65	−12.97	8.31	−0.40
8.36	−18.61	8.239	−14.900	7.459	−10.670	9.710	−57.820	$n_O \rightarrow R$(3p’,3s’’)	8.14	5.530	8.73	14.34	8.36	−18.02
8.42	9.91	8.309	3.460	7.502	2.008	9.897	17.430	$\sigma \rightarrow R$(3s’)	8.16	−6.280	8.75	−5.89	8.42	10.31
8.46	−9.31							$n_O \rightarrow R$(3p’’)	8.19	14.100				
8.57	0.02							$n_O \rightarrow R$(3s’’’)	8.30	−3.080				
8.60	−2.65							$\sigma \rightarrow R$(3p)	8.35	−13.900				
8.67	2.34							$n_O \rightarrow R$(3p’,3s’’)	8.50	3.260				
9.04	1.80													
9.18	−5.07													
9.22	6.54													
9.29	13.36													
9.38	−3.00													

[$]Calculations for the basis sets: 6-31+G*, 6-31++G**, aug-cc-pVDZ, aug-cc-pVTZ, aug-cc-pV5Z, SNSD and SNST are not reported for brevity.

Table S5c.

Comparison of our TD-DFT calculated vertical excitation energies (VE) and corresponding rotatory strengths (*R*) with the previous literature. Only the studies where at least 5 excitation energies were calculated are cited and where available the transition assignments are stated.

Present study		Cukras *et al*. 2016 (*47*)					
TD-DFT/CAM-B3LYP (aug-cc-pvQZ), *R*-PO		Hartree–Fock (t-aug-cc-pVDZ), *R*-PO		TD-DFT/CAM-B3LYP (t-aug-cc-pVDZ), *R*-PO		TD-DFT/B3LYP (t-aug-cc-pVDZ), *R*-PO	
VE (eV)	*R* (10^{-40} cgs)	VE (eV)	R_{length} (10^{-40} cgs)	VE (eV)	R_{length} (10^{-40} cgs)	VE (eV)	R_{length} (10^{-40} cgs)
7.16	−15.88	8.831	−1.009	7.101	−14.047	6.442	−16.326
7.49	−7.04	8.980	4.706	7.365	−8.042	6.836	7.493
7.58	8.69	9.214	−19.796	7.515	8.717	6.866	−2.007
7.74	7.33	9.265	−10.746	7.671	6.089	6.899	5.471
7.88	10.64	9.366	−4.041	7.851	9.508	7.214	−1.080
8.32	0.15	9.514	−12.327	8.244	−9.597	7.273	0.616
8.36	−18.61	9.648	10.261	8.279	9.733	7.280	−0.250
8.42	9.91	9.870	6.773	8.309	−10.258	7.284	0.911
8.46	−9.31	9.997	−2.728	8.349	0.740	7.302	6.634
8.57	0.02	10.160	7.693	8.373	−5.869	7.313	0.243
8.60	−2.65						
8.67	2.34						
9.04	1.80						
9.18	−5.07						
9.22	6.54						
9.29	13.36						
9.38	−3.00						

REFERENCES AND NOTES

1 B. A. McGuire, P. B. Carroll, R. A. Loomis, I. A. Finneran, P. R. Jewell, A. J. Remijan, G. A. Blake, Discovery of the interstellar chiral molecule propylene oxide (CH_3CHCH_2O). *Science* **352**, 1449–1452 (2016).

2 J. R. Brandt, F. Salerno, M. J. Fuchter, The added value of small-molecule chirality in technological applications. *Nat. Rev. Chem.* **1**, 1–12 (2017).

3 F. Lancia, A. Ryabchun, N. Katsonis, Life-like motion driven by artificial molecular machines. *Nat. Rev. Chem.* **3**, 536–551 (2019).

4 T. Leigh, P. Fernandez-Trillo, Helical polymers for biological and medical applications. *Nat. Rev. Chem.* **4**, 291–310 (2020).

5 Z. Liu, H. Du, J. Li, L. Lu, Z.-Y. Li, N. X. Fang, Nano-kirigami with giant optical chirality. *Sci. Adv.* **4**, eaat4436 (2018).

6 G. Long, R. Sabatini, M. I. Saidaminov, G. Lakhwani, A. Rasmita, X. Liu, E. H. Sargent, W. Gao, Chiral-perovskite optoelectronics. *Nat. Rev. Mater.* **5**, 423–439 (2020).

7 R. Naaman, Y. Paltiel, D. H. Waldeck, Chiral molecules and the electron spin. *Nat. Rev. Chem.* **3**, 250–260 (2019).

8 G. Qu, A. Li, C. G. Acevedo-Rocha, Z. Sun, M. T. Reetz, The crucial role of methodology development in directed evolution of selective enzymes. *Angew. Chem. Int. Ed.* **59**, 13204–13231 (2020).

9 G. F. Joyce, G. M. Visser, C. A. A. van Boeckel, J. H. van Boom, L. E. Orgel, J. van Westrenen, Chiral selection in poly(C)-directed synthesis of oligo(G). *Nature* **310**, 602–604 (1984).

10 W. A. Bonner, The origin and amplification of biomolecular chirality. *Orig. Life Evol. Biosph.* **21**, 59–111 (1991).

11 F. C. Frank, On spontaneous asymmetric synthesis. *Biochim. Biophys. Acta* **11**, 459–463 (1953).

12 M. Klussmann, T. Izumi, A. J. White, A. Armstrong, D. G. Blackmond, Emergence of solution-phase homochirality via crystal engineering of amino acids. *J. Am. Chem. Soc.* **129**, 7657–7660 (2007).

13 D. K. Kondepudi, R. J. Kaufman, N. Singh, Chiral symmetry breaking in sodium chlorate crystallization. *Science* **250**, 975–976 (1990).

14 M. Quack, How important is parity violation for molecular and biomolecular chirality? *Angew. Chem. Int. Ed.* **41**, 4618–4630 (2002).

15 J. B. Ribo, J. Crusats, F. Sagues, J. Claret, R. Rubires, Chiral sign induction by vortices during the formation of mesophases in stirred solutions. *Science* **292**, 2063–2066 (2001).

16 G. L. Rikken, E. Raupach, Enantioselective magnetochiral photochemistry. *Nature* **405**, 932–935 (2000).

17 R. A. Rosenberg, M. Abu Haija, P. J. Ryan, Chiral-selective chemistry induced by spin-polarized secondary electrons from a magnetic substrate. *Phys. Rev. Lett.* **101**, 178301 (2008).

18 K. Soai, S. Osanai, K. Kadowaki, S. Yonekubo, T. Shibata, I. Sato, *d*- and *l*-Quartz-promoted highly enantioselective synthesis of a chiral organic compound. *J. Am. Chem. Soc.* **121**, 11235–11236 (1999).

19 G. Tranter, Parity-violating energy differences of chiral minerals and the origin of biomolecular homochirality. *Nature* **318**, 172–173 (1985).

20 F. Vester, T. Ulbricht, H. Krauch, Optische Aktivität und die Paritätsverletzung im β-Zerfall. *Naturwissenschaften* **46**, 68–68 (1959).

21 P. De Marcellus, C. Meinert, M. Nuevo, J.-J. Filippi, G. Danger, D. Deboffle, L. Nahon, L.

Le Sergeant d'Hendecourt, U. J. Meierhenrich, Non-racemic amino acid production by ultraviolet irradiation of achiral interstellar ice analogs with circularly polarized light. *Astrophys. J. Lett.* **727**, L27 (2011).

22 J. J. Flores, W. A. Bonner, G. A. Massey, Asymmetric photolysis of (RS)-leucine with circularly polarized ultraviolet light. *J. Am. Chem. Soc.* **99**, 3622–3625 (1977).

23 U. J. Meierhenrich, L. Nahon, C. Alcaraz, J. H. Bredehöft, S. V. Hoffmann, B. Barbier, A. Brack, Asymmetric vacuum UV photolysis of the amino acid leucine in the solid state. *Angew. Chem. Int. Ed.* **44**, 5630–5634 (2005).

24 C. Meinert, S. V. Hoffmann, P. Cassam-Chenaï, A. C. Evans, C. Giri, L. Nahon, U. J. Meierhenrich, Photonenergy-controlled symmetry breaking with circularly polarized light. *Angew. Chem. Int. Ed.* **53**, 210–214 (2014).

25 H. Nishino, A. Kosaka, G. A. Hembury, F. Aoki, K. Miyauchi, H. Shitomi, H. Onuki, Y. Inoue, Absolute asymmetric photoreactions of aliphatic amino acids by circularly polarized synchrotron radiation: Critically pH-dependent photo behavior. *J. Am. Chem. Soc.* **124**, 11618–11627 (2002).

26 W. L. Noorduin, A. A. C. Bode, M. van der Meijden, H. Meekes, A. F. van Etteger, W. J. P. van Enckevort, P. C. M. Christianen, B. Kaptein, R. M. Kellogg, T. Rasing, E. Vlieg, Complete chiral symmetry breaking of an amino acid derivative directed by circularly polarized light. *Nat. Chem.* **1**, 729–732 (2009).

27 B. Norden, Was photoresolution of amino acids the origin of optical activity in life? *Nature* **266**, 567–568 (1977).

28 J. A. L. Bel, Sur les relations qui existent entre les formules atomiques des corps organiques et le pouvoir rotatoire de leurs dissolutions. *Bull. Soc. Chim. Paris* **22**, 337–347 (1874).

29 N. P. Huck, W. F. Jager, B. De Lange, B. L. Feringa, Dynamic control and amplification of molecular chirality by circular polarized light. *Science* **273**, 1686–1688 (1996).

30 J.-Y Kim, J. Yeom, H. Calcaterra, G. Zhao, P. Zhang, N. Kotov, Assembly of gold nanoparticles into chiral superstructures driven by circularly polarized light. *J. Am. Chem. Soc.* **141**, 11739–11744 (2019).

31 A. D. Garcia, C. Meinert, H. Sugahara, N. C. Jones, S. V. Hoffmann, U. J. Meierhenrich, The astrophysical formation of asymmetric molecules and the emergence of a chiral bias. *Life* **9**, 29 (2019).

32 I. Myrgorodska, C. Meinert, Z. Martins, L. Le Sergeant d'Hendecourt, U. J. Meierhenrich, Molecular chirality in meteorites and interstellar ices, and the chirality experiment on board the ESA cometary Rosetta mission. *Angew. Chem. Int. Ed.* **54**, 1402–1412 (2015).

33 H. K. Bisoyi, Q. Li, Light-directed dynamic chirality inversion in functional self-organized helical superstructures. *Angew. Chem. Int. Ed.* **55**, 2994–3010 (2016).

34 C. He, G. Yang, Y. Kuai, S. Shan, L. Yang, J. Hu, D. Zhang, Q. Zhang, G. Zou, Dissymmetry enhancement in enantioselective synthesis of helical polydiacetylene by application of superchiral light. *Nat. Commun.* **9**, 5117 (2018).

35 H. Kagan, A. Moradpour, J. F. Nicoud, G. Balavoine, G. Tsoucaris, Photochemistry with circularly polarized light. Synthesis of optically active hexahelicene. *J. Am. Chem. Soc.* **93**, 2353–2354 (2002).

36 S. T. Wu, Z. W. Cai, Q. Y. Ye, C. H. Weng, X. H. Huang, X. L. Hu, C. C. Huang, N. F. Zhuang, Enantioselective synthesis of a chiral coordination polymer with circularly polarized visible laser. *Angew. Chem. Int. Ed.* **53**, 12860–12864 (2014).

37 J. Lu, Y. Xue, K. Bernardino, N. N. Zhang, W. R. Gomes, N. S. Ramesar, S. Liu, Z. Hu, T. Sun, A. F. de Moura, N. A. Kotov, K. Liu, Enhanced optical asymmetry in supramolecular chiroplasmonic assemblies with long-range order. *Science* **371**, 1368–1374 (2021).

38 L. Wan, J. Wade, F. Salerno, O. Arteaga, B. Laidlaw, X. Wang, T. Penfold, M. J. Fuchter, A. J. Campbell, Inverting the handedness of circularly polarized luminescence from light-emitting polymers using film thickness. *ACS Nano* **13**, 8099–8105 (2019).

39 M. D. Ward, J. Wade, X. Shi, J. Nelson, A. J. Campbell, M. J. Fuchter, Highly selective high-speed circularly polarized photodiodes based on π-conjugated polymers. *Adv. Opt. Mater.* **10**, 2101044 (2021).

40 G. Pescitelli, L. Di Bari, N. Berova, Conformational aspects in the studies of organic compounds by electronic circular dichroism. *Chem. Soc. Rev.* **40**, 4603–4625 (2011).

41 W. Kuhn, E. Braun, Photochemische Erzeugung optisch aktiver Stoffe. *Naturwissenschaften* **17**, 227–228 (1929).

42 W. Kuhn, E. Knopf Photochemische Erzeugung optisch aktiver Stoffe. *Naturwissenschaften* **18**, 183 (1930).

43 C. Meinert, J. H. Bredehöft, J. J. Filippi, Y. Baraud, L. Nahon, F. Wien, N. C. Jones, S. V. Hoffmann, U. J. Meierhenrich, Anisotropy spectra of amino acids. *Angew. Chem. Int. Ed.* **51**, 4484–4487 (2012).

44 A. Breest, P. Ochmann, F. Pulm, K. H. Gödderz, M. Carnell, J. Hormes, Experimental circular dichroism and VUV spectra of substituted oxiranes and thiiranes. *Mol. Phys.* **82**, 539–551 (1994).

45 M. Carnell, Experimental and quantum-theoretical investigation of the circular dichroism spectrum of R-methyloxirane. *Chem. Phys. Lett.* **180**, 477–481 (1991).

46 D. Cohen, M. Levi, H. Basch, A. Gedanken, Excited electronic states of optically active substituted ethylene oxides:(−)-(S)-2-methyloxirane and (−)-(S, S)-2, 3-dimethyloxirane. *J. Am. Chem. Soc.* **105**, 1738–1742 (1983).

47 J. Cukras, J. Kauczor, P. Norman, A. Rizzo, G. L. J. A. Rikken, S. Coriani, A complex-polarization-propagator protocol for magneto-chiral axial dichroism and birefringence dispersion. *Phys. Chem. Chem. Phys.* **18**, 13267–13279 (2016).

48 M. M. Rafiee Fanood, I. Powis, M. H. Janssen, Chiral asymmetry in the multiphoton ionization of methyloxirane using femtosecond electron–ion coincidence imaging. *J. Phys.*

Chem. A **118**, 11541–11546 (2014).

49 L. Alagna, S. di Fonzo, T. Prosperi, S. Turchini, P. Lazzeretti, M. Malagoli, R. Zanasi, C. R. Natoli, P. J. Stephens, Random phase approximation calculations of K-edge rotational strengths of chiral molecules: Propylene oxide. *Chem. Phys. Lett.* **223**, 402–410 (1994).

50 M. Hodecker, M. Biczysko, A. Dreuw, V. Barone, Simulation of vacuum UV absorption and electronic circular dichroism spectra of methyl oxirane: The role of vibrational effects. *J. Chem. Theory Comput.* **12**, 2820–2833 (2016).

51 D. Kroner, Laser-driven electron dynamics for circular dichroism in mass spectrometry: From one-photon excitations to multiphoton ionization. *Phys. Chem. Chem. Phys.* **17**, 19643–19655 (2015).

52 C. Meinert, A. D. Garcia, J. Topin, N. C. Jones, M. Diekmann, R. Berger, L. Nahon, S. V. Hoffmann, U. J. Meierhenrich, Amino acid gas phase circular dichroism and implications for the origin of biomolecular asymmetry. *Nat. Commun.* **13**, 502 (2022).

53 S. Pizzarello, C. T. Yarnes, Chiral molecules in space and their possible passage to planetary bodies recorded by meteorites. *Earth Planet. Sci. Lett.* **496**, 198–205 (2018).

54 T. Yanai, D. P. Tew, N. C. Handy, A new hybrid exchange–correlation functional using the Coulomb-attenuating method (CAM-B3LYP). *Chem. Phys. Lett.* **393**, 51–57 (2004).

55 T. Miyahara, J.-y. Hasegawa, H. Nakatsuji, Circular dichroism and absorption spectroscopy for three-membered ring compounds using Symmetry-Adapted Cluster-Configuration Interaction (SAC-CI) method. *Bull. Chem. Soc. Jpn.* **82**, 1215–1226 (2009).

56 J. Neugebauer, E. J. Baerends, M Nooijen, J. Autschbach, Importance of vibronic effects on the circular dichroism spectrum of dimethyloxirane. *J. Chem. Phys.* **122**, 234305 (2005).

57 J. R. Cronin, S. Pizzarello, Enantiomeric excesses in meteoritic amino acids. *Science* **275**, 951–955 (1997).

58 D. P. Glavin, J. P. Dworkin, Enrichment of the amino acid L-isovaline by aqueous alteration on CI and CM meteorite parent bodies. *Proc. Natl. Acad. Sci. U.S.A.* **106**, 5487–5492 (2009).

59 G. Cooper, A. C. Rios, Enantiomer excesses of rare and common sugar derivatives in carbonaceous meteorites. *Proc. Natl. Acad. Sci. U.S.A.* **113**, E3322–3331 (2016).

60 J. Bailey, A. Chrysostomou, J. H. Hough, T. M. Gledhill, A. McCall, S. Clark, F., Ménard, M. Tamura, Circular polarization in star- formation regions: Implications for biomolecular homochirality. *Science* **281**, 672–674 (1998).

61 M. Buschermohle, D. C. B. Whittet, A. Chrysostomou, J. H. Hough, P. W. Lucas, A. J. Adamson, B. A. Whitney, M. J. Wolff, An extended search for circularly polarized infrared radiation from the OMC-1 region of Orion. *Astrophys. J.* **624**, 821–826 (2005).

62 A. Chrysostomou, P. W. Lucas, J. H. Hough, Circular polarimetry reveals helical magnetic fields in the young stellar object HH 135-136. *Nature* **450**, 71–73 (2007).

63 J. Kwon, M. Tamura, P. W. Lucas, J. Hashimoto, N. Kusakabe, R. Kandori, Y. Nakajima, T. Nagayama, T. Nagata, J. H. Hough, Near-infrared circular polarization images of NGC 6334-V. *Astrophys. J.* **765**, L6 (2013).

64 A. Bergantini, M. J. Abplanalp, P. Pokhilko, A. I. Krylov, C. N. Shingledecker, E. Herbst, R. I. Kaiser, A combined experimental and theoretical study on the formation of interstellar propylene oxide (CH_3CHCH_2O)—A chiral molecule. *Astrophys. J.* **860**, 108 (2018).

65 D. R. Paulson, A. S. Murray, D. Bennett, E. Mills Jr., V. O. Terry, S. D. Lopez, Photochemistry of epoxides. 3. Direct irradiation of propylene oxide in the gas phase. *J. Org. Chem.* **42**, 1252–1254 (1977).

66 J. C. Forbes, J. Alves, D. N. C. Lin, A solar system formation analogue in the Ophiuchus star-forming complex. *Nat. Astron.* **5**, 1009–1016 (2021).

67 J. R. Cronin, S. Pizzarello, Amino acid enantiomer excesses in meteorites: Origin and

significance. *Adv. Space Res.* **23**, 293–299 (1999).

68 S. Pizzarello, J. R. Cronin, Non-racemic amino acids in the Murray and Murchison meteorites. *Geochim. Cosmochim. Acta* **64**, 329–338 (2000).

69 A. J. Miles, S. V. Hoffmann, Y. Tao, R. W. Janes, B. A. Wallace, Synchrotron radiation circular dichroism (SRCD) spectroscopy: New beamlines and new applications in biology. *Spectroscopy* **21**, 245–255 (2007).

70 A. J. Miles, R. W. Janes, A. Brown, D. T. Clarke, J. C. Sutherland, Y. Tao, B. A. Wallace, S. V. Hoffmann, Light flux density threshold at which protein denaturation is induced by synchrotron radiation circular dichroism beamlines. *J. Synchrotron Radiat.* **15**, 420–422 (2008).

71 F. Pulm, J. Schramm, J. Hormes, S. Grimme, S. D. Peyerimhoff, Theoretical and experimental investigations of the electronic circular dichroism and absorption spectra of bicyclic ketones. *Chem. Phys.* **224**, 143–155 (1997).

72 A. C. Evans, C. Meinert, J. H. Bredehoeft, C. Giri, N. C. Jones, S. V. Hoffmann, U. J. Meierhenrich, Anisotropy spectra for enantiomeric differentiation of biomolecular building blocks. *Top. Curr. Chem.* **341**, 271–299 (2013).

73 J. Bloino, M. Biczysko, F. Santoro and V. Barone, General approach to compute vibrationally resolved one-photon electronic spectra. *J. Chem. Theory Comput.* **6**, 1256–1274 (2010).

74 A. Rizzo, O. Vahtras, *Ab initio* study of excited state electronic circular dichroism. Two prototype cases: Methyl oxirane and R-(+)-1,1′-bi(2-naphthol). *J. Chem. Phys.* **134**, 244109 (2011).
